# Expression of Elongase‐ and Desaturase‐Encoding Genes Shapes the Cuticular Hydrocarbon Profiles of Honey Bees

**DOI:** 10.1111/mec.17716

**Published:** 2025-03-06

**Authors:** Daniel Sebastián Rodríguez‐León, Thomas Schmitt, María Alice Pinto, Markus Thamm, Ricarda Scheiner

**Affiliations:** ^1^ University of Würzburg, Biocenter Department of Animal Ecology and Tropical Biology Würzburg Germany; ^2^ University of Würzburg Biocenter, Department of Behavioral Physiology and Sociobiology Würzburg Germany; ^3^ CIMO, LA SusTEC Instituto Politécnico de Bragança Bragança Portugal

**Keywords:** CHC biosynthesis, chemical ecology, gene expression, social insects

## Abstract

Most terrestrial insects have a layer of cuticular hydrocarbons (CHCs) protecting them from desiccation and mediating chemical communication. The composition of these hydrocarbons is highly plastic and changes during their lifetime and with environmental conditions. How these changes in CHC composition are achieved is largely unknown. CHC profiles of *Apis mellifera* honey bees vary among castes, task groups and subspecies adapted to different climates. This makes *A. mellifera* an excellent model for studying the molecular mechanism underlying CHC biosynthesis. We correlated the expression of specific elongase‐ and desaturase‐encoding genes with the CHC composition in bees performing different social tasks in two highly divergent *A. mellifera* subspecies. Elongases are enzymes that lengthen the hydrocarbon chain, while desaturases introduce double bonds in it. We evaluated the hypothesis that the expression of the genes encoding these enzymes determines CHC profiles of the worker bees. Our results revealed that the specificity of desaturases and elongases shapes the CHC profiles of worker bees performing different social tasks. Expression of the desaturase‐encoding gene LOC100576797 and the elongase‐encoding gene LOC550828 seemed to be strongly associated with the abundance of compounds that were characteristic of the CHC profile of nurse bees. In contrast, the compounds that characterised the CHC profiles of the forager bees seemed to be associated with the desaturase‐encoding gene LOC551527 and the elongase‐encoding gene LOC409638. Our data shed light on the genetic basis for task‐specific CHC composition differences in social hymenopterans and paved the ground for unravelling the genetic underpinning of CHC biosynthesis.

## 1 Introduction

Cuticular hydrocarbons (CHCs) protect insects from water loss and mediate inter‐ and intraspecific communication (Blomquist et al. 1987; Gibbs 1998; Howard and Blomquist 2005). The biological function of the CHC layer is determined by its composition (Blomquist et al. 1987; Howard and Blomquist 2005). For example, its ability to act as a desiccation barrier depends on the aggregation of its constituent hydrocarbons (Gibbs 1995; Gibbs 2002; Menzel et al. 2019). The n‐alkanes aggregate denser than methyl‐branched or unsaturated hydrocarbons due to stronger van der Waals forces (Gibbs 1998; Gibbs and Pomonis 1995). The aggregation of hydrocarbons also increases with their chain length (Gibbs and Pomonis 1995). The denser the aggregation of the hydrocarbons, the more waterproof the CHC layer is (Gibbs 1995; Gibbs 2002; Howard and Blomquist 2005). But a dense aggregation of hydrocarbons also increases the viscosity of the CHC layer, thereby limiting hydrocarbon diffusion and interindividual CHC exchanges and constraining the function of CHCs as communication cues (Gibbs 1995; Gibbs 2002; Gibbs and Pomonis 1995; Menzel et al. 2019).

CHCs are primarily synthesised by oenocytes, specialised secretory cells associated with the fat bodies of insects (Makki et al. 2014; Moris et al. 2023; Schal et al. 1998; Wigglesworth 1933). The CHC biosynthetic pathway is closely related to fatty acid metabolism. It is initiated with the synthesis of fatty acyl‐coenzyme A (acyl‐CoA) from the binding of malonyl‐CoA units to acetyl‐CoA (Blomquist and Bagnères 2010; Howard and Blomquist 2005). Along this pathway, several enzymes modify the fatty acyl‐CoA to ultimately produce the different CHCs (Chung and Carroll 2015; Holze et al. 2021). Some of these enzymes contribute to the elongation of the hydrocarbon chain (elongases) (Cinti et al. 1992; Denic and Weissman 2007), while others introduce unsaturations (double bonds) into the hydrocarbon chain (desaturases) (Dallerac et al. 2000; Takahashi et al. 2001). The abundance and richness of CHC compounds on the cuticle of an insect are determined by the diversity and expression of the enzymes participating in this biosynthetic pathway (Gu et al. 1997; Juárez et al. 1992; Qiu et al. 2012; Reed et al. 1995). The expression of desaturase‐encoding genes influences the diversity and abundance of unsaturated hydrocarbons (e.g., alkenes and alkadienes), while the expression of elongase‐encoding genes influences the diversity and abundance of compounds with specific chain lengths. However, little is known about how the expression of genes encoding enzymes of the same type (e.g., different elongases and desaturases) contributes to the variability of insect CHC composition.

A great example of how gene expression differences can lead to differential phenotypes can be found in social insects, making them excellent models for studying the relationship between gene expression and complex phenotypes (Hunt et al. 2010; Hunt et al. 2013; Smith et al. 2008). In honey bees ( *Apis mellifera* ), the CHC profiles of the workers differ according to their age, task performance and subspecies (Kather et al. 2011; Rodríguez‐León et al. 2024; Vernier et al. 2019). The CHC profiles of nurse bees are characterised by a higher abundance of unsaturated hydrocarbons and longer chain lengths compared to forager bees (Kather et al. 2011; Rodríguez‐León et al. 2024). These two social task groups typically differ in age. Young workers typically care for the brood and the queen (nurse bees), while the oldest workers leave the colony to collect pollen and nectar (forager bees) (Huang and Robinson 1996). Because the expression of specific elongase‐ and desaturase‐encoding genes varies with age (Vernier et al. 2019), the differences in the CHC composition between nurses and foragers assumedly respond to a developmental regulatory process.

The differences in the CHC profiles of nurse and forager bees are present in different *A. mellifera* subspecies (Rodríguez‐León et al. 2024). *A. mellifera* subspecies have divergent evolutionary histories, leading to both genetic and phenotypic differences (Dogantzis et al. 2021; Garnery et al. 1992; Ruttner et al. 1978; Ruttner 1988). Different subspecies have evolved distinct CHC profiles, exhibiting both quantitative and qualitative differences in their composition (Rodríguez‐León et al. 2024). This remarkable variability of CHC profiles makes *A. mellifera* an excellent model for studying the molecular mechanisms underlying CHC biosynthesis. A recent study elucidated a clear role of genes encoding specific elongases and desaturases in the CHC biosynthesis of honey bee workers (Moris et al. 2023). A knockdown of these genes resulted in compositional changes in CHC profiles, aligning with the expected function of the respective enzyme type. However, the role of desaturase and elongase gene expression in shaping CHC profiles has remained unclear.

Here, we correlated the expression of elongase‐ and desaturase‐encoding genes in nurse and forager honey bees of two highly divergent subspecies, *Apis mellifera carnica* and *Apis mellifera iberiensis* , with their CHC profile composition. We hypothesised that the shift in CHC composition during the transition from nursing to foraging is driven by changes in the expression of specific CHC biosynthesis‐related genes. These changes in gene expression should be preserved in both subspecies. We also hypothesised that the subspecies‐specific CHC compositions of honey bee workers are influenced by differences in the expression of specific CHC biosynthesis‐related genes between subspecies.

## 2 Materials and Methods

We performed a common garden experiment to rule out the impact of the local environment on the subspecies‐specific differences in the expression of CHC biosynthesis‐related genes and CHC profiles of the bees. For this, we maintained queen‐right colonies of *A. m. carnica* and *A. m. iberiensis* in the departmental apiary of the University of Würzburg (Germany). Although these subspecies are both native to Europe, they belong to the most evolutionarily divergent lineages of *A. mellifera* (Wallberg et al. 2014). *A. m. carnica* belongs to the Eastern European C‐lineage, whereas *A. m. iberiensis* belongs to the Western European M‐lineage (Ruttner 1988). *A. m. carnica* is native to the Central and Southeastern parts of Europe, but it has been introduced worldwide, and it is now the dominant subspecies in Germany (Maul and Hähnle 1994). *A. m. iberiensis* is native to the Iberian Peninsula, where it experienced historical secondary contact with the African A lineage (Chávez‐Galarza et al. 2015; de la Rúa et al. 2009; Han et al. 2012; Ruttner 1988).

The colonies were assembled on 2 June 2021, using artificial swarms obtained from *A. m. carnica* colonies and mated queens from both subspecies. *A. m. carnica* queens were obtained from local apiaries in Würzburg (Germany), and *A. m. iberiensis* queens were obtained from local apiaries in Bragança (Portugal). The *A. m. iberiensis* queens were shipped according to valid veterinary legislation and were registered in the TRACES‐Database. Ethical approval and collection permission for the present study are not applicable under local regulations since the experiments involved unprotected honey bees from research apiaries.

Honey bee workers were collected between the 22nd and 23rd of September 2021, between 9 AM and noon. Ten nurse bees and 10 forager bees were taken from two colonies per subspecies, for a total of 80 worker bees. The 40 nurse bees were picked while poking their heads into a brood cell for at least 10 s. The 40 forager bees were picked while landing at the hive entrance. Worker bees with noticeable pollen loads were collected to ensure accurate task classification. The workers were collected into individual vials and transported to the lab on ice, where they were killed by storing them at −80°C. The collected bees were preserved at −80°C until the extraction of their CHCs.

### 2.1 CHC Composition Analysis

The bees were defrosted and immersed in hexane for only 2 min to extract their CHCs and avoid RNA degradation. Immediately after the extraction with hexane, the bees were dissected to remove their guts and separate the abdomen from the rest of the body. The dissected abdomens were preserved separately in 963 μL of RNA‐later, stored at 4°C for 48 h, and then at −20°C until their RNA was extracted. The CHC extracts were stored at −20°C.

The CHC extracts were analysed via gas chromatography/mass spectrometry (GC/MS) by injecting 1 μL of each extract into an Agilent 7890A Series GC System coupled to an Agilent 5975C Mass Selective Detector (Agilent Technologies, Waldbronn, Germany) operating in electron impact ionisation mode at 70 eV and a source temperature of 230°C. The split/splitless injector was operated in splitless mode for 1 min at 300°C. Separation of compounds was performed on a J&W DB‐5 fused silica capillary column (30 m × 0.25 mm ID, df = 0.25 μm, J&W, Folsom, California, USA) with a temperature program starting at 60°C and increasing by 5°C per minute to 300°C, which was held for 10 min. Helium served as a carrier gas with a constant flow of 1 mL per minute.

The chromatograms were analysed using the data analysis software package ‘MassHunter Workstation Software—Qualitative Analysis Navigator’ (version B.08.00; Agilent Technologies Inc. 2016). The areas of the peaks were determined by integration using the ‘Agile2’ parameter‐less integrator, with the peak filters set to 0% of the area of the largest peak. The integration results were aligned regarding the retention time (RT) of the peaks for all the samples of each colony and task‐performance (e.g., nurse or forager bees) group. The differences in performance of the total ion counts were corrected based on n‐alkane analytical standard solutions (04070‐1ML and 04071‐5ML, Sigma‐Aldrich). The aligned group data sets were merged by the Kováts retention index (RI) and compound identity of the peaks. Compounds that represented less than 0.01% of the total ion count of a sample, and compounds detected in < 50% of the samples within a group, were excluded from the analysis. Only the compounds identified as alkanes, alkenes, alkadienes, or methyl‐branched alkanes were considered in the analysis. The different hydrocarbons in the extracts were identified based on their diagnostic ions and RIs. Double bond positions of monounsaturated hydrocarbons (alkenes) were identified by dimethyl disulfide derivatization (Carlson et al. 1989). The abundances of the compounds were quantified for each sample as the proportion (%) that the area of the corresponding peaks represented from the sum of the area of all the peaks included in the analysis.

Additionally, we calculated the relative abundance of both alkenes and alkadienes, as well as the mean chain length in the CHC profile of each bee and compared them between tasks and between subspecies. The relative abundances of the alkenes and alkadienes (mono‐ and diunsaturated hydrocarbons, respectively) were calculated as the sum of the relative abundances of all the individual alkenes or alkadienes found in the CHC extract of a bee. The mean chain length corresponds to the weighted mean of the chain length of all the compounds found in the CHC profile of each bee, using the relative abundance of the compounds as their weights.

### 2.2 Gene Expression Analysis

Since the oenocytes are associated with the fat bodies, and these are in the abdomen, we chose to analyse the expression of the desaturase‐ and elongase‐encoding genes in the abdomen of the bees.

The GenUpTM total RNA kit (Biotechrabbit, Henningsdorf, Germany) was used to extract the total RNA from each of the dissected abdomens, following the manufacturer's instructions. Along the RNA extraction procedure, a DNase I digestion step was performed by adding 30 μL DNase mix containing 30 U RNase‐free DNase I (Lucigen Corporation, Middleton, USA) together with the corresponding buffer, after the binding of the RNA to the Mini Filter RNA. The total RNA concentration was determined photometrically, and the extracted RNA was diluted in DEPC‐H_2_O to a concentration of 100 ng/μL and stored at −80°C until the synthesis of the cDNA. A total of 400 ng of RNA was used for cDNA synthesis with the cDNA synthesis kit 331475S/L (Biozym, Hessisch Oldendorf, Germany). The resulting cDNA solution was diluted up to a total volume of 180 μL with DEPC‐H_2_O and stored at −20°C.

The expression of two desaturase‐encoding genes (*Des1* and *Des2*; Table S1) and two elongase‐encoding genes (*Elo1* and *Elo2*; Table S1) was determined via quantitative Polymerase Chain Reaction (qPCR). These four genes were chosen as they have been shown to affect CHC biosynthesis in honey bees (Moris et al. 2023). Individual cDNAs were analysed in triplicate for each gene using the SYBR Green BlueMix qPCR kit (Biozym, Hessisch Oldendorf, Germany) and a Rotor‐Gene Q (Qiagen, Hilden, Germany). The following cycling conditions were set up for every qPCR run: 2 min at 95°C, 40 cycles with 5 s at 95°C and 30 s at 60°C. A melting curve was recorded by increasing the temperature from 60°C to 95°C in steps of 1°C every 5 s. Four qPCR runs were performed per gene, including the reference genes (*Rpl10* and *Rpl19*; Table S1), for a total of 16 qPCR runs. Each qPCR run included five samples per task and subspecies, with all samples analysed in triplicate. All samples of both subspecies within a qPCR run corresponded to the same hive, and the same combination of samples per hive per run was used for the four genes. The expression of the four genes was quantified relative to the reference genes, following the qBase algorithm (Hellemans et al. 2007). Cycle threshold (*Ct*) values of sample replicates that deviated by more than 2 from the median *Ct* value of the corresponding sample were considered technical outliers and discarded from the calculation of the relative expression of the corresponding gene.

### 2.3 Data Analysis

We compared the relative abundance of alkenes and alkadienes, as well as the mean chain length, in the CHC profile of the bees between tasks (nurse bees vs. forager bees) and subspecies (*A. m. carnica* vs. *A. m. iberiensis*). We fitted quantile regression models for the 50% quantile, considering both task and subspecies as independent variables (predictors). The interaction between task and subspecies was only considered for the model cases where it was significant (i.e., the relative abundance of the alkenes).

To evaluate the difference in gene expression of the four assessed genes between tasks and subspecies, we performed a bootstrapped gamma generalised linear model (GLM), with a logarithmic link function, over 10,000 resamples. Inference was made with the bootstrapped marginal means, model coefficients (standardised effect sizes), and their 95% confidence intervals (CIs), which were calculated via the percentile intervals method. Due to the use of the logarithmic link function, the model coefficients were transformed by exponentiation. Thus, they represent the proportional difference in gene expression to the reference group (task: nurse bees; subspecies: *A. m. carnica*).

We evaluated the correlation of the relative expression of both desaturase‐encoding genes to the relative abundance of both alkenes and alkadienes, as well as to the relative abundance of every unsaturated hydrocarbon in the CHC profiles of the bees. Similarly, for the elongase‐encoding genes, we evaluated the correlation of their relative expression to the mean chain length of the CHCs of the bees, as well as to the relative abundance of every compound in the CHC profiles of the bees. All correlations were calculated using Spearman's correlation index. In addition, we performed a bi‐dimensional Non‐Metric Multidimensional Scaling (NMDS) using the Bray–Curtis dissimilarity index to assess the dissimilarity between samples regarding the composition of their CHC profiles. Then, for each of the four assessed genes, we plotted on the NMDS the compounds with a positive (> 0.4) or negative (< −0.4) correlation towards the expression of the corresponding gene.

The data analysis was performed using R v4.4.0 (R Core Team 2024) and RStudio IDE v2024.9.1.394 (Posit Team 2024). Data wrangling and plotting operations were done using the packages tidyverse v2.0.0 (Wickham et al. 2019), ggtext v0.1.2 (Wilke and Wiernik 2022), gghalves v0.1.4 (Tiedemann 2022), ragg v1.3.2 (Pedersen and Shemanarev 2023), and patchwork v1.2.0 (Pedersen 2023). The processing of the integrated chromatograms' data (i.e., RT wise alignment, RI calculation, abundance correction, peak filtering and calculation of the relative abundance of compounds) was done using the packages analyzeGC v0.2.1 (Rodríguez‐Leon 2023a) and GCalignR v1.0.7 (Ottensmann et al. 2018). The relative expression of the genes of interest was calculated using the package easyqpcr2 v0.1.0 (Rodríguez‐Leon 2023b). The bootstrapped GLMs and quantile regression models were fitted with the packages tidymodels v1.2.0 (Kuhn and Wickham 2020) and quantreg v5.98 (Koenker 2023), respectively. The marginal means were estimated using the package emmeans v1.10.3 (Lenth 2023). The NMDS was performed using the package vegan v2.6.6.1 (Oksanen et al. 2022). Spearman's correlation indexes and their 95% confidence intervals were calculated using the package confintr v1.0.2 (Mayer 2023).

## 3 Results

### 3.1 CHC Composition Differs Between Bees Performing Different Tasks and Between Subspecies

**FIGURE 1:**
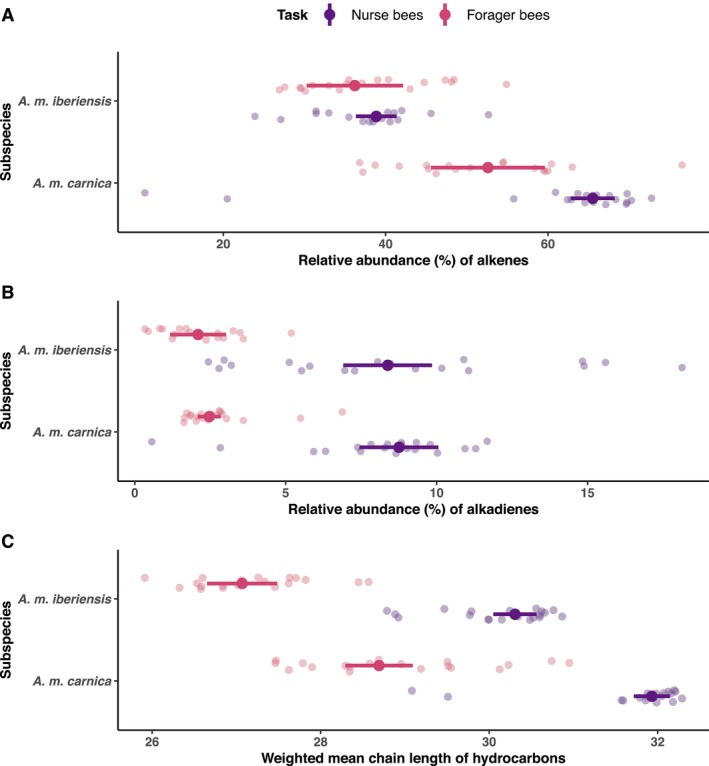
Task and subspecies‐related differences in the cuticular hydrocarbon (CHC) compositions of honey bee workers (nurse and forager bees) of two subspecies (*A. m. carnica* and *A. m. iberiensis*). The figure is divided into three plots, each corresponding to the results of a quantile regression analysis on the task‐ and subspecies‐related differences in a compositional trait of the CHC profiles of the bees. (A) Relative abundance of mono‐unsaturated hydrocarbons (alkenes). (B) Relative abundance of di‐unsaturated CHCs (alkadienes). (C) Mean chain length of the hydrocarbons. Point intervals represent the model prediction for the median and its 95% confidence interval. The raw data is visualised with transparent points, which correspond to the measured amount in the CHC profile of every bee.

*A. m. carnica* nurse bees exhibited a higher abundance of both alkenes (95% CI: 3.016, 22.790) and alkadienes (95% CI: 4.512, 8.045), as well as longer chain length hydrocarbons (95% CI: 2.700, 3.777) than forager bees (Figure 1 and Figure S1). In *A. m. iberiensis*, the nurse bees displayed a higher abundance of alkadienes (95% CI: 4.521, 8.045) and longer chain length hydrocarbons (95% CI: 2.700, 3.777) than forager bees. Subspecies did not affect the abundance of alkadienes (Figure 1B and Figure S1B; 95% CI: −1.200, 0.131). However, the CHC profile of *A. m. carnica* contained a higher abundance of alkenes and longer chain length hydrocarbons than that of *A. m. iberiensis*, in both nurse (alkenes abundance—95% CI: 21.813, 31.531; mean chain length—95% CI: 1.190, 2.059) and forager (alkenes abundance—95% CI: 4.314, 28.505; mean chain length—95% CI: 1.190, 2.059) bees (Figure 1 and Figure S1).

### 3.2 Nurse and Forager Bees Differ in the Expression of Desaturase‐ and Elongase‐Encoding Genes

**FIGURE 2:**
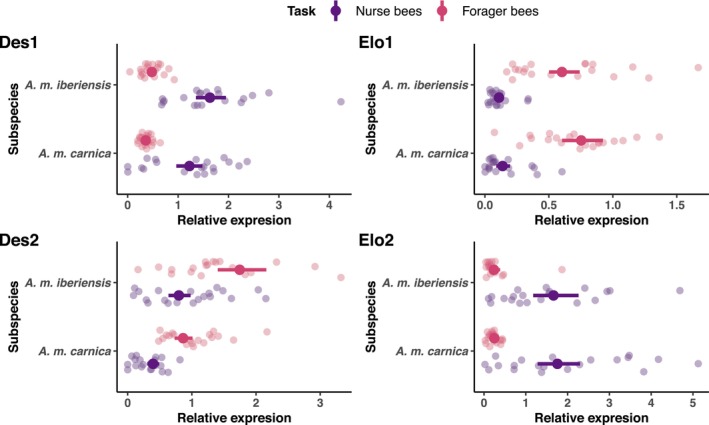
Relative expression of cuticular hydrocarbon (CHC) biosynthesis‐related genes in honey bee workers (nurse and forager bees) of two subspecies (*A. m. carnica* and *A. m. iberiensis*) genes. The figure is divided into four plots, each corresponding to a gene. Point intervals represent the median of the bootstrapped generalise linear model (GLM) prediction for the mean relative expression of the genes and its 95% confidence interval. The raw data is visualised with transparent points, which correspond to the relative gene expression value that was measured for every honey bee worker.

The expression of the two desaturase‐encoding genes differed between nurse and forager bees in both subspecies (Figure 2 and Figure S2). Forager bees expressed *Des1* 0.295 times as much as nurse bees (95% CI: 0.240, 0.363). Conversely, the expression of *Des2* in the forager bees was 2.209 times higher than that in the nurse bees (95% CI: 1.722, 2.819).

Nurse and forager bees also differed in the expression of both elongase‐encoding genes in both subspecies (Figure 2 and Figure S2). *Elo1* was expressed 5.586 times more by the forager bees than by the nurse bees (95% CI: 4.053, 7.774). On the other hand, the expression of *Elo2* in the foragers was 0.145 times that of the nurse bees (95% CI: 0.095, 0.214).

### 3.3 Subspecies Differ in the Expression of Desaturase‐ but Not Elongase‐Encoding Genes

*A. m. iberiensis* elicited a higher expression of both desaturase‐encoding genes than *A. m. carnica*, in nurse and forager bees (Figure 2 and Figure S2). The expression of *Des1* was 1.346 times higher in *A. m. iberiensis* than in *A. m. carnica* (95% CI: 1.092, 1.658). Moreover, the expression of *Des2* was 2.039 times higher in *A. m. iberiensis* than in *A. m. carnica* (95% CI: 1.594, 2.593). Subspecies did not differ in the expression of *Elo1* (95% CI: 0.589, 1.135) or *Elo2* (95% CI: 0.639, 1.426). This pattern was observed for both nurse and forager bees (Figure 2 and Figure S2).

### 3.4 Correlation Between Gene Expression and CHC Composition

**FIGURE 3:**
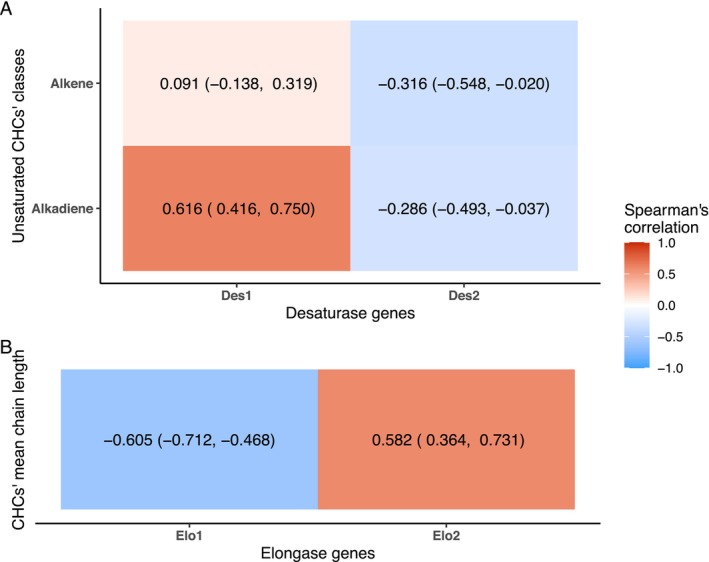
Correlation of the expression of cuticular hydrocarbon (CHC) biosynthesis‐related genes and CHC composition in honey bee workers (nurse and forager bees) of two subspecies (*A. m. carnica* and *A. m. iberiensis*). (A) Correlation between the relative expression of both desaturase‐encoding genes and the relative abundance of alkenes and alkadienes (%). (B) Correlation between the relative expression of both elongase‐encoding genes and the weighted mean chain length of the CHCs. The estimated Spearman's correlation index is shown besides its 95% confidence interval (lower limit, upper limit).

Bees with a higher relative expression of *Des1* exhibited a higher abundance of alkadienes in their CHC profiles (Figure 3A; 95% CI: 0.416, 0.750). In contrast, the relative expression of *Des2* was negatively correlated with the abundance of both alkenes (95% CI: −0.548, −0.020) and alkadienes (95% CI: −0.493, −0.037).

**FIGURE 4:**
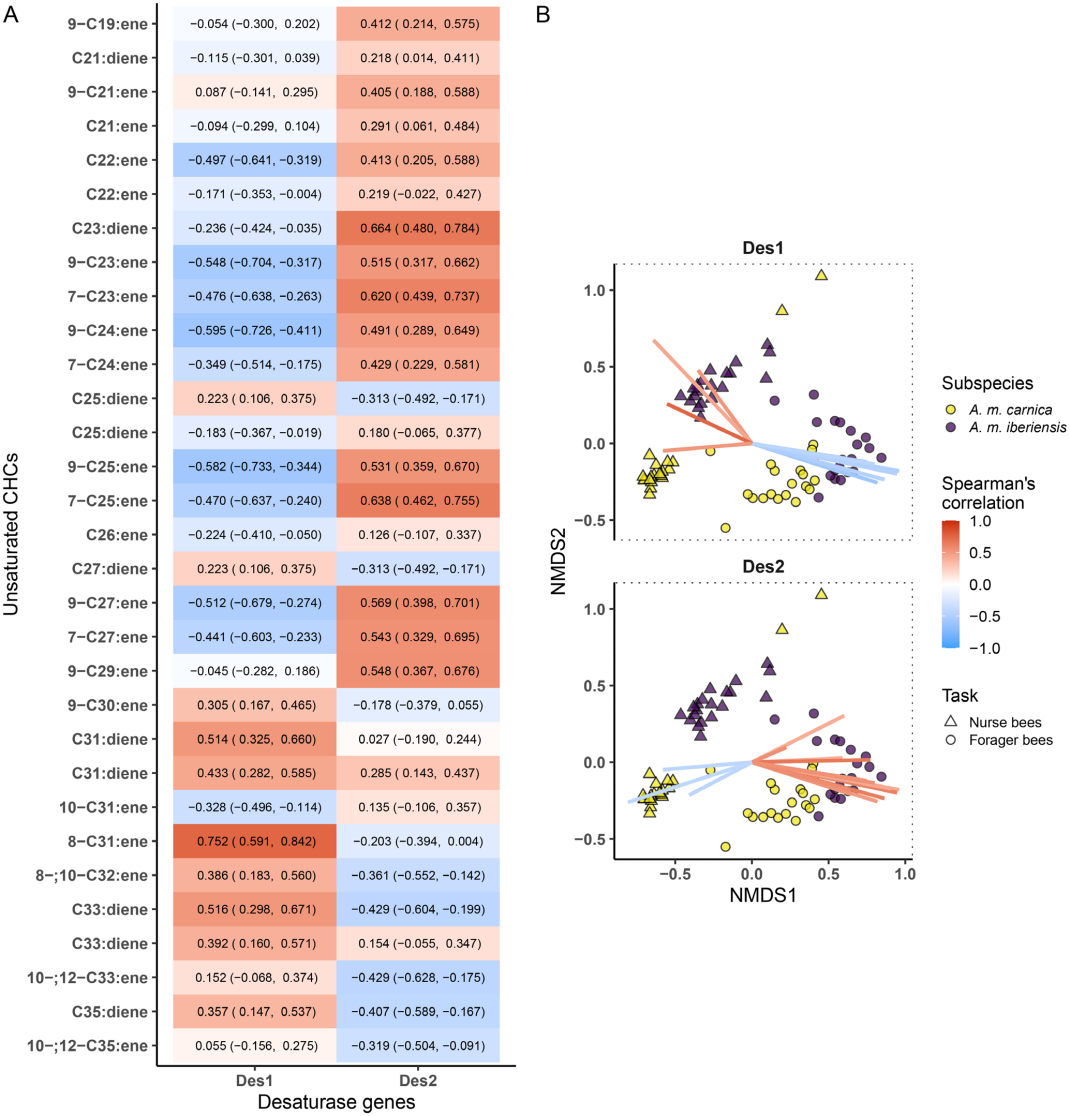
Correlation between the expression of desaturase‐encoding genes and the cuticular hydrocarbon (CHC) composition of honey bee workers (nurse and forager bees) of two subspecies (*A. m. carnica* and *A. m. iberiensis*). (A) Correlation between the relative expression of both desaturase‐encoding genes and the relative abundance (%) of hydrocarbons. The hydrocarbons are sorted from shorter chain length (top) to longer chain length (bottom). The estimated Spearman's correlation index is shown besides its 95% confidence interval (lower limit, upper limit). (B) Bi‐dimensional Non‐metric Multidimensional Scaling (NMDS) analysis on a dissimilarity matrix of the CHC profiles of honey bee workers. Ordination stress: 0.083. The plot is divided into facets, each depicting the same NMDS but only illustrating the coordinates of the CHCs correlated with the expression of a specific desaturase gene. The coloured lines illustrate the coordinates of the CHCs. The estimated correlation between hydrocarbons' abundance (%) and the expression of the corresponding gene is indicated by the colour of the line (red: Positive. blue: Negative). Only hydrocarbons with a correlation higher than −0.4 (negative) or 0.4 (positive) are shown.

Bees with a higher expression of *Des1* tended to have a higher abundance of unsaturated hydrocarbons with chain lengths over 29 carbon atoms in their CHC profiles (Figure 4A). Bees with a higher expression of *Des2* displayed higher abundances of unsaturated compounds with up to 29 carbon atoms in their CHC profiles. The compounds that positively correlated with the expression of *Des1* were more abundant in the CHC profile of nurse bees, while those negatively correlating with the expression of *Des1* were more abundant in the CHC profiles of forager bees (Figure 4B). *A. m. iberiensis* nurse bees had a higher abundance of the hydrocarbons that positively correlated with the expression of *Des1* than the nurse bees of *A. m. carnica*. In contrast, forager bees of *A. m. iberiensis* exhibited a higher abundance of the hydrocarbons that negatively correlated with the expression of *Des1* than their *A. m. carnica* counterparts. In the case of *Des2*, forager bees displayed a higher abundance of the hydrocarbons that were positively correlated with its expression. Nurse bees, in turn, presented a higher abundance of the hydrocarbons that negatively correlated with the expression of *Des2*. *A. m. iberiensis* forager bees presented a higher abundance of the hydrocarbons that positively correlated with the expression of *Des2* than the forager bees of *A. m. carnica*. The nurses of *A. m. carnica*, on the contrary, displayed a higher abundance of the hydrocarbons that negatively correlated with the expression of *Des2* than their *A. m. iberiensis* counterparts.

**FIGURE 5:**
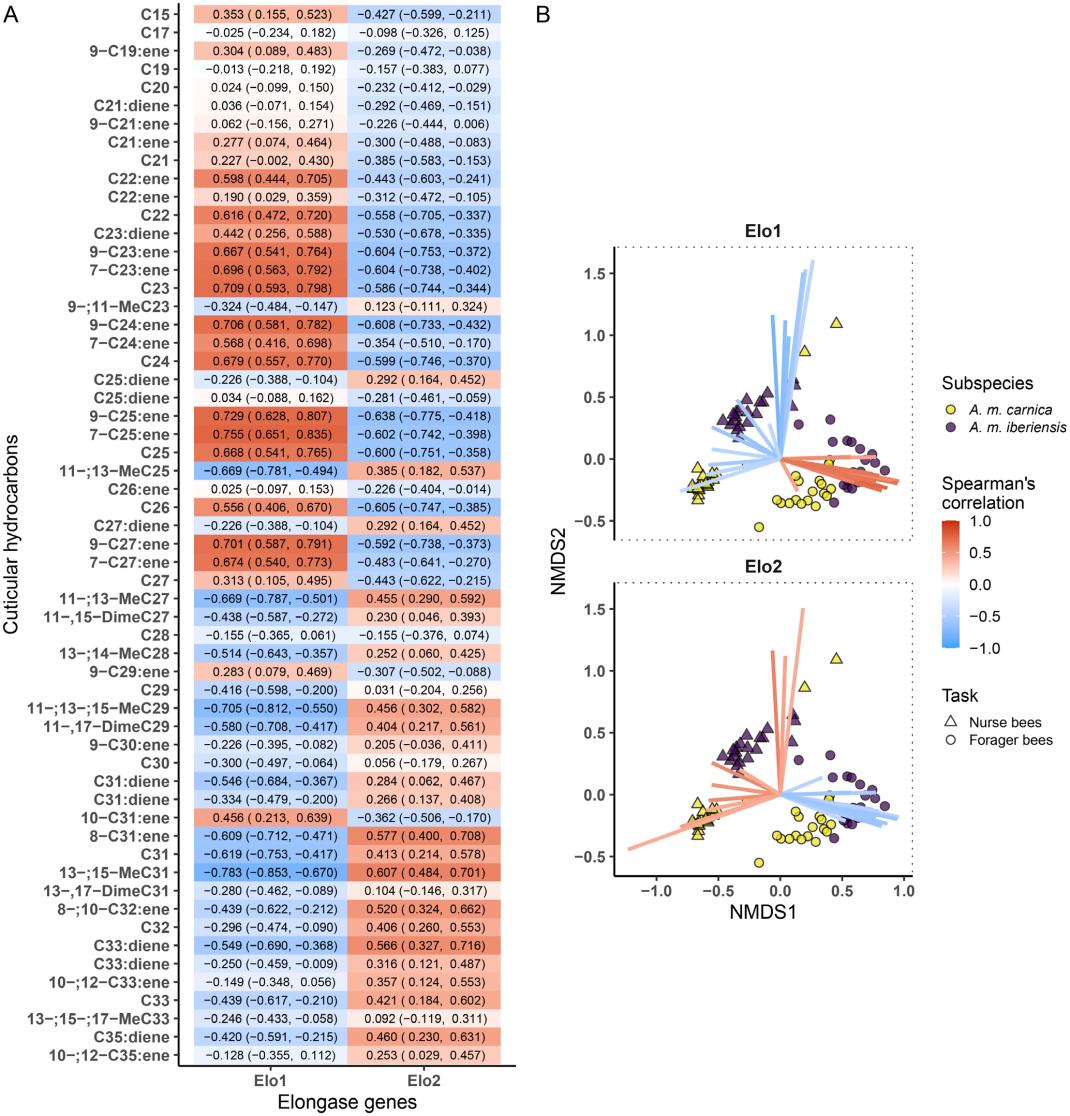
Correlation between the expression of elongase encoding genes and the cuticular hydrocarbon (CHC) compositions of honey bee workers (nurse and forager bees) of two subspecies (*A. m. carnica* and *A. m. iberiensis*). (A) Correlation between the relative expression of both elongase genes and the relative abundance (%) of hydrocarbons. The hydrocarbons are sorted, top‐to‐bottom, from shorter to longer chain length. The estimated Spearman's correlation index is shown besides its 95% confidence interval (lower limit, upper limit). (B) Bi‐dimensional Non‐metric Multidimensional Scaling (NMDS) analysis on a dissimilarity matrix of the CHC profiles of honey bee workers. Ordination stress: 0.083. The plot is divided into facets, each depicting the same NMDS but only illustrating the coordinates of the CHCs correlated with the expression of a specific elongase gene. The coloured lines illustrate the coordinates of the CHCs. The estimated correlation between hydrocarbons' abundance (%) and the expression of the corresponding gene is indicated by the colour of the line (red: Positive. blue: Negative). Only hydrocarbons with a correlation higher than −0.4 (negative) or 0.4 (positive) are shown.

Bees with a higher expression of *Elo1* tended to have shorter compounds in their CHC profiles (Figure 3B; 95% CI: −0.712, −0.468). More specifically, the expression of *Elo1* correlated positively with the abundance of compounds with chain lengths between 22 and 27 carbon atoms and negatively with the abundance of longer compounds (Figure 5A). On the contrary, bees with a higher expression of *Elo2* had the tendency to exhibit CHC profiles with longer compounds (Figure 3B; 95% CI: 0.364, 0.731). The higher the expression of *Elo2*, the higher the abundance of the longer compounds (≥ 27 carbon atoms). In turn, compounds with chain lengths shorter than 27 carbon atoms tended to be less abundant in the CHC profiles of the bees with a higher expression of *Elo2*.

The expression of both elongase‐encoding genes strongly correlated with the abundance of task‐specific compounds (Figure 5B). *Elo1* correlated positively with the hydrocarbons that were more abundant in the CHC profiles of forager bees and negatively with those that were more abundant in the CHC profiles of nurse bees. *A. m. iberiensis* forager bees displayed a higher abundance of the compounds that were positively correlated with the expression of *Elo1* compared to *A. m. carnica* forager bees. This difference between subspecies was less evident among nurse bees. However, most of the hydrocarbons that correlated negatively with the expression of *Elo1* were more abundant in the *A. m. iberiensis* nurse bees than in their *A. m. carnica* counterparts.

The compounds that correlated positively with the expression of *Elo2* were more abundant in nurse bees compared to forager bees. On the contrary, forager bees displayed a higher abundance of the compounds that correlated negatively with the expression of *Elo2* than nurse bees. Here, the differences between the two subspecies seem to be evident only between the forager bees, with *A. m. iberiensis* displaying a higher abundance of the compounds that were negatively correlated with the expression of *Elo2* than *A. m. carnica*.

## 4 Discussion

### 4.1 Nurse and Forager Bees Differ in Their CHC Compositions

Our data revealed a different CHC composition between nurse and forager bees. Nurse bees had longer chain hydrocarbons and more abundant unsaturated compounds in their CHC profile compared to forager bees. This difference was stereotypically consistent for two evolutionary divergent *A. mellifera* subspecies. Furthermore, it suggests that the CHC layer of forager bees protects them better against desiccation compared to nurse bees (Menzel, Blaimer, et al. 2017; Menzel, Schmitt, et al. 2017; Menzel et al. 2018). A similar difference has been described for the workers inside and outside the nest of other social insects (Martin and Drijfhout 2009; Wagner et al. 1998, 2001). This is thought to respond to the higher desiccation pressure faced by outside workers (e.g., forager bees) compared to those inside the nest (e.g., nurse bees), due to their exposure to weather extremes (e.g., temperature and humidity) (Kather et al. 2011; Martin and Drijfhout 2009; Rodríguez‐León et al. 2024; Wagner et al. 1998, 2001). Moreover, this difference in CHC composition between workers inside and outside the nest might serve as cues to inform other workers about the tasks they perform (Greene and Gordon 2003; Martin and Drijfhout 2009).

Honey bee workers exhibit an age‐dependent division of labour, transitioning from nursing to foraging as they age. This transition is accompanied by a series of physiological changes (Johnson 2008; Elekonich et al. 2001; Reim and Scheiner 2014; Scheiner et al. 2014; Scheiner et al. 2017; Schilcher and Scheiner 2023; Siegel et al. 2013). The observed differences in the CHC composition of nurse and forager bees could therefore be influenced by shifts in the expression of CHC biosynthesis‐related genes that occur along their task transition.

### 4.2 Underlying Mechanism of the Task‐Related CHC Composition Shift in Honey Bees

The four examined genes (*Des1*, *Des2*, *Elo1* and *Elo2*) strongly differed in their expression between nurse and forager bees. *Des1* and *Elo2* were up‐regulated in nurse bees and down‐regulated in forager bees, while the opposite pattern was observed for *Des2* and *Elo1*. A limitation of our study is that it does not allow us to disentangle the effect of the age of the bees and the task they perform, due to their age‐dependent division of labour. However, the division of labour of honey bees is flexible and responds to the needs of the colony, allowing individual bees to transition from nursing to foraging at different ages (Beck et al. 2023; Huang and Robinson 1996; Johnson and Frost 2012; Winston and Fergusson 1985; Woyciechowski and Moroń 2009). In fact, by artificially manipulating the colony, forager bees can be induced to revert to nurse bees, both behaviorally and physiologically (Robinson et al. 1992). In this study, the difference in the expression of CHC biosynthesis‐related genes between nurse and forager bees correlates with their different CHC compositions. Specifically, the expression of *Des1* and *Elo2* correlated positively with the abundance of compounds that were characteristic of nurse bees. The expression of *Des2* and *Elo1*, on the other hand, correlated positively with the abundance of compounds that were characteristic of forager bees. Therefore, it is plausible that the observed differences in the expression of CHC biosynthesis‐related genes between nurse and forager bees respond to regulatory changes associated with task transition. Furthermore, these results open the door for future studies to decipher this yet unknown regulatory mechanism. For example, a future study could evaluate if the expression of these genes is influenced by juvenile hormone, vitellogenin and/or octopamine, considering the role these substances play in the regulation of the age‐dependent division of labour of honey bees (Schilcher and Scheiner 2023). Intriguingly, the regulatory mechanism underlying such changes appears to be conserved across evolutionary lineages, as is suggested by the consistent correlation between task‐specific CHC profiles and the expression of these genes in both *A. mellifera* subspecies.

### 4.3 *A. m. carnica* and *A. m. iberiensis* Differ in CHC Composition

*A. m. carnica* displayed longer compounds and a higher abundance of unsaturated compounds in their CHC profiles than *A. m. iberiensis*. These results suggest that *A. m. iberiensis* is better adapted to a higher drought stress than *A. m. carnica* (Menzel, Blaimer, et al. 2017; Menzel, Schmitt, et al. 2017; Menzel et al. 2018). However, differences in CHC composition between *A. mellifera* subspecies have been suggested to respond to their divergent evolutionary histories and genetic drift, as they do not seem to consistently correlate with the climatic differences (i.e., temperature and precipitation) between their native distributions (Rodríguez‐León et al. 2024). Therefore, the adaptive difference in CHC composition we observed between *A. m. carnica* and *A. m. iberiensis* is likely specific to this particular comparison. These two subspecies belong to different *A. mellifera* evolutionary lineages and their natural ranges encompass very different environmental conditions (Chávez‐Galarza et al. 2015; de la Rúa et al. 2009; Han et al. 2012; Ruttner 1988). As our study was a common garden experiment, we attribute the observed differences in the CHC composition between *A. m. carnica* and *A. m. iberiensis* to innate factors, leading us to expect an innate difference in the expression of CHC biosynthesis‐related genes between the two subspecies.

### 4.4 *A. mellifera* Subspecies Differences Highlight CHC Biosynthesis Complexity

*A. m. iberiensis* exhibited a higher expression of both desaturase‐encoding genes (*Des1* and *Des2*) than *A. m. carnica*. In contrast, the expression of both elongase‐encoding genes (*Elo1* and *Elo2*) did not differ between subspecies. These findings suggest that the expression of *Des1* and *Des2* among *A. mellifera* subspecies would respond to their evolutionary divergence, but not the expression of *Elo1* and *Elo2*. Since we performed a common garden experiment, the similarity in the expression of *Elo1* and *Elo2* by the bees of both subspecies could indicate an influence of the local (external) environment on the expression of these two elongase‐encoding genes. Furthermore, we cannot rule out a possible influence of the CHC profile signature left by the *A. m. carnica* workers of the artificial swarms originally used to assemble the hives of both subspecies in the common garden experiment. Social insects form a gestalt odour profile (Lenoir et al. 1999; Van Zweden and D'Ettorre 2010). This is part of the social colony environment, which in *A. mellifera* can even influence the CHC composition of unrelated foster workers (Vernier et al. 2019). In this sense, the CHC composition of the initial workers of the colonies might have induced some similarities in the CHC composition between subspecies, which could be related to the expression of the two elongase‐encoding genes (*Elo1* and *Elo2*). However, our results do not discard the possibility that the expression of these genes responds to intrinsic factors that are not related to the subspecies.

Nevertheless, among the genes assessed here, only the expression of *Des2* suggests a plausible mechanism underlying the differences in the CHC composition between *A. m. carnica* and *A. m. iberiensis*. The expression of this gene is positively correlated with the abundance of hydrocarbons with chain lengths of up to 29 carbon atoms. In consequence, the higher expression of *Des2* in *A. m. iberiensis* compared to *A. m. carnica* could contribute to the shorter mean chain length of hydrocarbons in the CHC profile of *A. m. iberiensis* compared to *A. m. carnica*. The expression of *Des1*, on the other hand, is correlated with the abundance of alkadienes, a feature not differing between subspecies. Similarly, while the CHC profile of *A. m. carnica* exhibited longer hydrocarbons compared to *A. m. iberiensis*, the expression of both elongase‐encoding genes (*Elo1* and *Elo2*) did not differ between subspecies. These discrepancies between the subspecific differences in CHC composition and the expression of the four studied genes evidence the complexity of CHC biosynthesis. Although several genes have been proposed to participate in CHC biosynthesis in honey bees, only a few have been mechanistically confirmed through targeted knock‐down experiments to directly influence CHC composition in worker bees (Falcón et al. 2014; Moris et al. 2023; Vernier et al. 2019). It is thus plausible that the observed subspecific differences in CHC composition stem from the activity of the enzymes encoded by other CHC biosynthesis‐related genes.

### 4.5 Differential Roles of Specific Genes in CHC Biosynthesis

Our findings suggest that *Des1* and *Des2* play distinct roles in CHC biosynthesis. The expression of *Des1* correlated positively with the total relative abundance of alkadienes in the CHC profile of the bees, while the expression of *Des2* correlated negatively with the abundance of both alkenes and alkadienes. We consider that this difference could stem from the specificities of the enzymes encoded by *Des1* and *Des2*. Different desaturases have been found to vary in their substrate specificities and, thereby, in how they influence CHC composition (Dallerac et al. 2000; Holze et al. 2021; Takahashi et al. 2001). In our experiments, the expression of the two desaturase‐encoding genes correlated with the abundance of different unsaturated hydrocarbons, supporting the above hypothesis. Moreover, the suggested difference in specificities between the enzymes encoded by these two genes explains the negative correlation between the expression of *Des2* and the abundance of both alkenes and alkadienes. In contrast to *Des1*, the expression of *Des2* was positively correlated with the abundance of unsaturated hydrocarbons with chain lengths of up to 29 carbon atoms. These compounds were the least abundant in the CHC profiles of honey bee workers, considering the mean chain length of the hydrocarbons in all analysed groups (nurse and forager bees of both *A. m. carnica* and *A. m. iberiensis*) (Figure 1C). These compounds therefore only contributed little to the total abundance of alkenes and alkadienes.

In the case of the elongase‐encoding genes, we found that *Elo1* expression negatively correlated with the mean chain length of the CHCs, unlike *Elo2*. While this *Elo1* pattern seems counterintuitive, as elongases catalyse the elongation of the hydrocarbon chain (Chung and Carroll 2015; Cinti et al. 1992; Jakobsson et al. 2006), this result can be explained by a differential specificity of the elongases encoded by the two genes. Elongases have been found to generally catalyse the biosynthesis of hydrocarbons with chain lengths of over 20 carbon atoms. Thus, the difference in specificity among elongases would explain the diversity of chain lengths in the CHC profiles of insects (Blomquist and Bagnères 2010; Denic and Weissman 2007; Holze et al. 2021). The expression of *Elo1* and *Elo2* correlated with the abundance of compounds within different chain length ranges, suggesting that both genes influence the synthesis of hydrocarbons with different chain lengths.

The diversity and regulation of the expression of CHC biosynthesis‐related genes are thought to influence the richness and abundance of compounds in the CHC profile of an insect (Blomquist and Bagnères 2010; Chung and Carroll 2015; Gu et al. 1997; Holze et al. 2021; Howard and Blomquist 2005; Juárez et al. 1992; Qiu et al. 2012; Reed et al. 1995). However, current knowledge of the differential roles of enzymes of the same type (e.g., specific desaturases) shaping CHC composition is limited. Our data shed light on this topic by revealing correlations between the expression of specific desaturase‐ and elongase‐encoding genes and CHC compositions in *A. mellifera* workers.

### 4.6 Key Insights and Future Directions

Our study reveals conserved correlations between CHC composition and expression of specific CHC biosynthesis‐related genes in honey bees. We provide evidence for a differential specificity among individual desaturases and elongases and propose that differences in the expression of their encoding genes contribute to shaping the CHC profiles of *A. mellifera* workers during their task transition from nursing to foraging. These results lay the ground for further studies aiming to unravel the genetic underpinning of CHC biosynthesis, in particular regarding task‐specific CHC composition differences among social hymenopterans. One limitation of this study is that we could not disentangle the effect of age and task in the expression of CHC biosynthesis‐related genes or the CHC profiles of the bees. Future studies should consider experimental designs that allow disentangling the effect of age and task, like the usage of single cohort colonies. We suggest further investigating *A. mellifera* subspecies to better understand the mechanisms underlying the CHCs diversity of honey bees. The complexity of the genetic basis underlying the difference in CHC composition among *A. mellifera* subspecies could reveal exciting insight into the molecular basis of CHC biosynthesis.

## Author Contributions

Conceptualization: D.S.R.‐L., T.S. and R.S.; Work design: D.S.R.‐L., M.T. and T.S.; data acquisition: D.S.R.‐L.; data analysis and interpretation: D.S.R.‐L., M.T., T.S., R.S. and M.A.P.; writing of original draft: D.S.R.‐L., T.S., R.S., M.A.P. and M.T.; reviewing and editing: D.S.R.‐L., M.T., R.S., M.A.P. and T.S.; supervision: T.S., R.S. and M.A.P.

## Conflicts of Interest

The authors declare no conflicts of interest.

## Supporting information


Data S1.

## Data Availability

The data and code used for this study can be accessed through DOI: https://doi.org/10.6084/m9.figshare.26831491.
