# Supplementary material for: Expression of Elongase‐ and Desaturase‐Encoding Genes Shapes the Cuticular Hydrocarbon Profiles of Honey Bees
Source: Mol Ecol. 2025 Mar 6;34(8):e17716. doi: 10.1111/mec.17716 (PMC11974489; doi:10.1111/mec.17716)
Supplement: Supplementary file 1 — Data S1. [file MEC-34-e17716-s001.docx]

Supporting information for:

Expression of elongase- and desaturase-encoding genes shapes the cuticular hydrocarbon profiles of honey bees

Daniel Sebastián Rodríguez-León^1,^ ^3,^ *

Thomas Schmitt^1^

María Alice Pinto^2^

Markus Thamm^3^

Ricarda Scheiner^3^

^1^University of Würzburg, Biocenter, Department of Animal Ecology and Tropical Biology, Am Hubland, 97074 Würzburg, Germany.

^2^ CIMO, LA SusTEC, Instituto Politécnico de Bragança, Campus de Santa Apolónia, 5300-253 , Bragança, Portugal

^3^University of Würzburg, Biocenter, Department of Behavioral Physiology and Sociobiology, Am Hubland, 97074 Würzburg, Germany.

*Corresponding author: [daniel.rodriguez@uni-wuerzburg.de](mailto:daniel.rodriguez@uni-wuerzburg.de) (DS Rodríguez-León).

Table S1: Oligonucleotide primers used in this study.

|  | Gene | Gene ID | Primer direction | Primer sequence (5'-3') |
| --- | --- | --- | --- | --- |
| Gene of interest | *Des1* | LOC100576797 | Reverse | TGATGATAATTATGCCATCCTTC |
|  |  |  | Forward | TGGTTGGTAAATTCTGCTGCT |
|  | *Des2* | LOC551527 | Reverse | GTAAGAGGTTGTTTGAAGAA |
|  |  |  | Forward | TTGGATTAACGGAACGAAGG |
|  | *Elo1* | LOC409638 | Reverse | CCAAACCACACTGACATTGG |
|  |  |  | Forward | TTCATGGACACGATCTTCTTTG |
|  | *Elo2* | LOC550828 | Reverse | TTATTACAAGGACAAATTGAACC |
|  |  |  | Forward | TTGGTTTGGCGTTAAGTTCA |
| Reference gene | *Rpl10* | LOC409589 | Reverse | CGTATCTTTGGATCAGGCACACC |
|  |  |  | Forward | CGATAAGAAACGTAAGTCAATATGGGGC |
|  | *Rpl19* | LOC724186 | Reverse | GCTTTGACGTGAGTTTGTATTTGCAA |
|  |  |  | Forward | GGGACTTCTAGGCTCCATCATGAG |


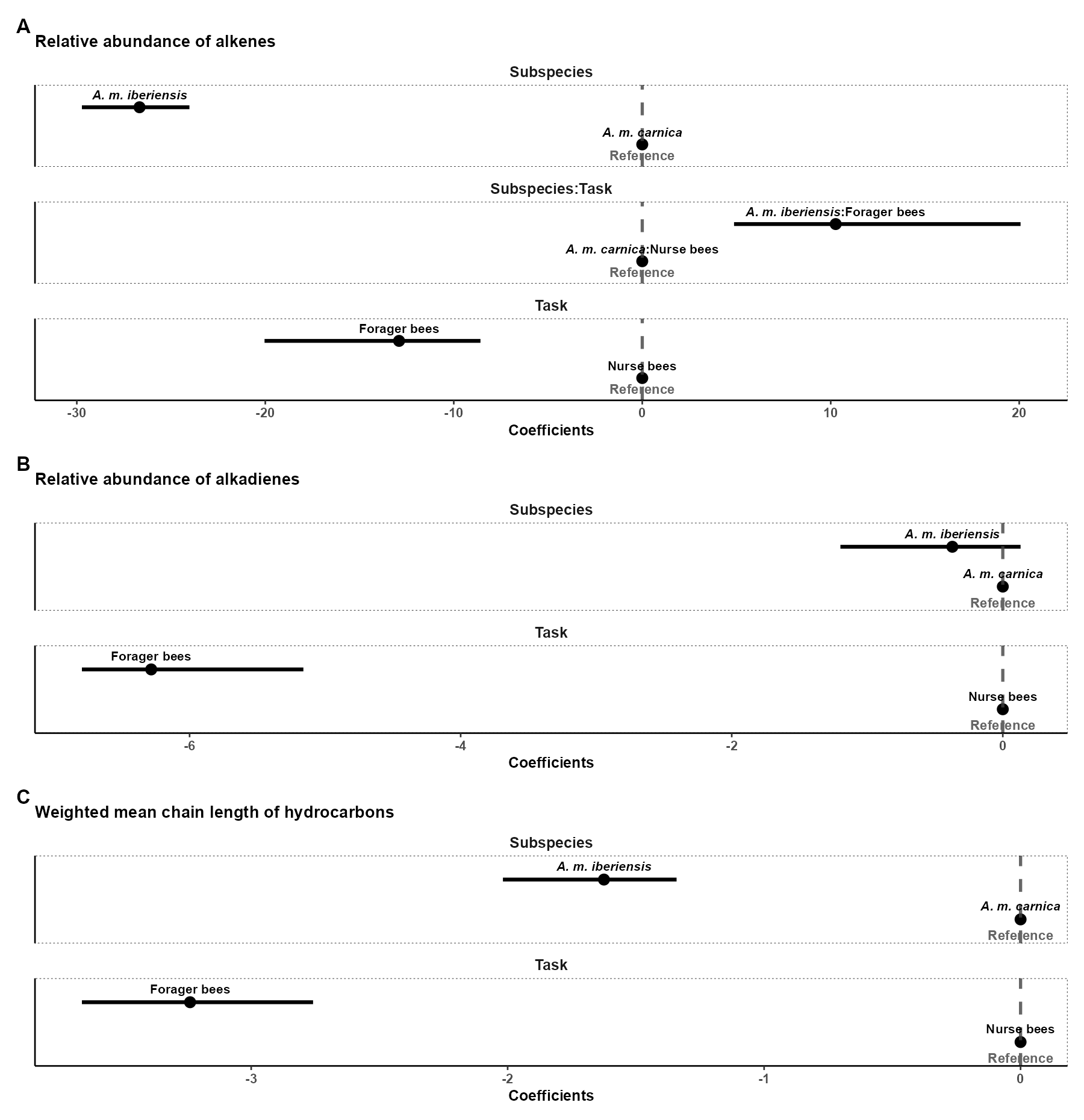


Figure S1: Quantile regression coefficients (effect sizes) for the task and subspecies-related differences in the cuticular hydrocarbon (CHC) composition of honey bee workers. The figure is divided into three plots, each corresponding to the results of a quantile regression (50$\text{\%}$ quantile) analysis on the task- and subspecies-related differences in a compositional trait of the CHC profile of honey bee workers. $\text{A)}$ Relative abundance of mono-unsaturated hydrocarbons (alkenes). $\text{B)}$ Relative abundance of di-unsaturated hydrocarbons (alkadienes). $\text{C)}$ Mean chain length of the CHCs. Each plot is divided into facets, which correspond to the categorical variables used as predictors for the model (task and subspecies) and their interaction (task:subspecies), if included. Point intervals represent the estimated difference to the reference group (task: nurse bees; subspecies: $\text{A. m. ca}\text{rnica}$; subspecies:task: $\text{A. m. carnica}$:nurse bees) and its 95$\text{\%}$ confidence intervals. The reference is visualized with a dashed grey line and an overlapping point for the corresponding group.


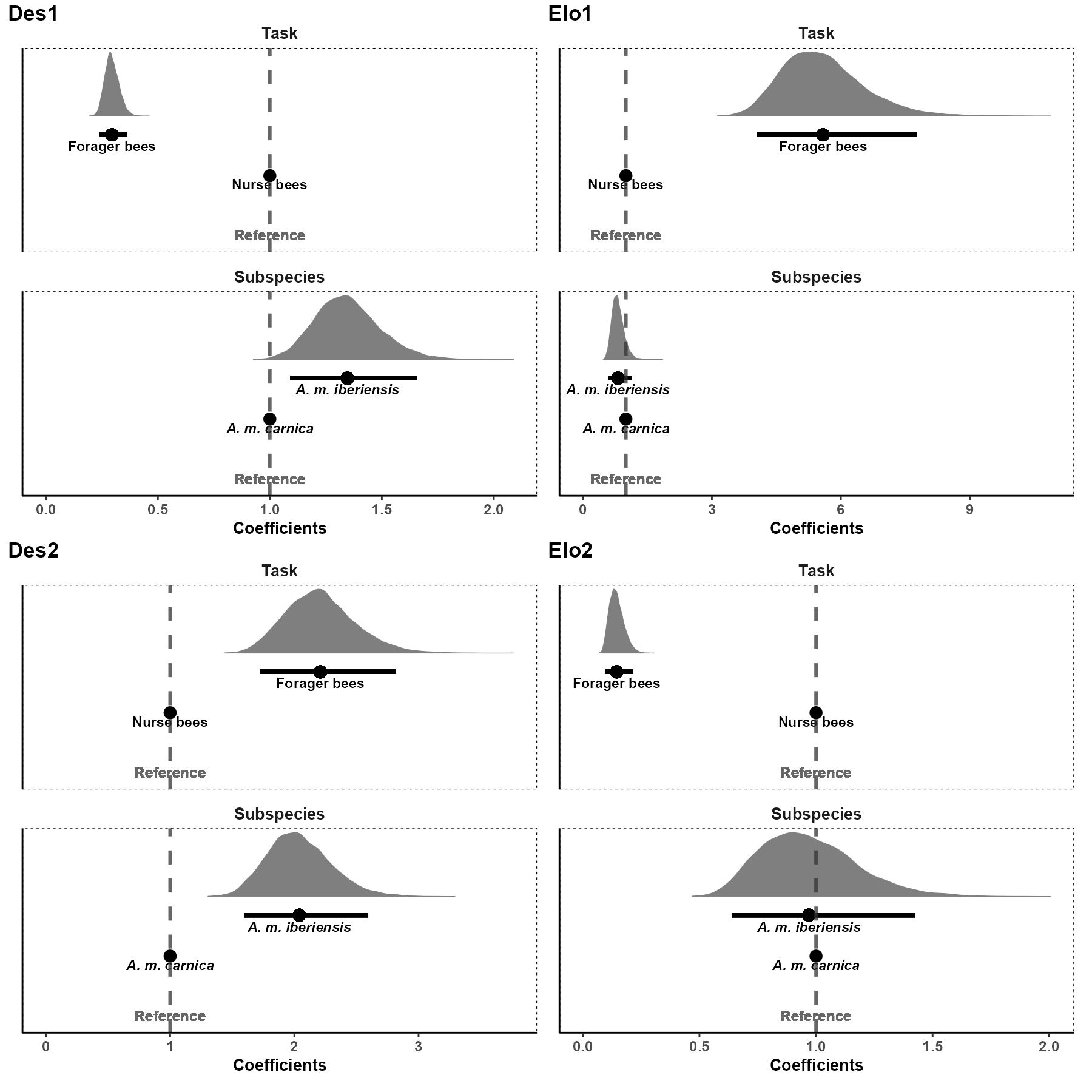


Figure S2: Bootstrapped generalized linear model (GLM) coefficients (effect sizes) for the difference in the relative expression of the cuticular hydrocarbon (CHC) biosynthesis-related genes between tasks and subspecies. The figure is divided into four plots, each corresponding to a gene. Each plot is divided into two facets, which correspond to the two categorical variables used as predictors for the model (task and subspecies). Shaded curves depict the distribution of the estimated GLM coefficients across the 10,000 bootstrap simulations. Point intervals represent the median of the bootstrapped GLM mean proportional difference in the gene expression to the reference group (task: nurse bees; subspecies: $\text{A. m. carnica}$) and its 95$\text{\%}$ confidence intervals. The reference is visualized with a dashed grey line and an overlapping point for the corresponding group.
